# The value of episodic, intensive blood glucose monitoring in non-insulin treated persons with type 2 diabetes: Design of the Structured Testing Program (STeP) Study, a cluster-randomised, clinical trial [NCT00674986]

**DOI:** 10.1186/1471-2296-11-37

**Published:** 2010-05-18

**Authors:** William Polonsky, Lawrence Fisher, Charles Schikman, Deborah Hinnen, Christopher Parkin, Zhihong Jelsovsky, Linda Amstutz, Matthias Schweitzer, Robin Wagner

**Affiliations:** 1 University of California, San Diego, and Behavioral Diabetes Institute, P.O Box 501866, San Diego, California, 92150, USA; 2 University of California, San Francisco, 400 Parnassus Avenue, San Francisco, California, 94122, USA; 3 North Shore University Health System, 9600 Gross Point Road, Skokie, Illinois, 60076, USA; 4 Mid America Diabetes Associates, 200 South Hillside, Wichita, Kansas, 67211, USA; 5 Health Management Resources, Inc., 11360 Royal Court, Carmel, Indiana, 46032, USA; 6 Biostat International, Inc., 14506A University Point Place, Tampa, Florida, 33613, USA; 7 Roche Diagnostics, 9115 Hague Road, Indianapolis, Indiana, 46250, USA

## Abstract

**Background:**

The value and utility of self-monitoring of blood glucose (SMBG) in non-insulin treated T2DM has yet to be clearly determined. Findings from studies in this population have been inconsistent, due mainly to design differences and limitations, including the prescribed frequency and timing of SMBG, role of the patient and physician in responding to SMBG results, inclusion criteria that may contribute to untoward floor effects, subject compliance, and cross-arm contamination. We have designed an SMBG intervention study that attempts to address these issues.

**Methods/design:**

The Structured Testing Program (STeP) study is a 12-month, cluster-randomised, multi-centre clinical trial to evaluate whether poorly controlled (HbA1c ≥ 7.5%), non-insulin treated T2DM patients will benefit from a comprehensive, integrated physician/patient intervention using structured SMBG in US primary care practices. Thirty-four practices will be recruited and randomly assigned to an active control group (ACG) that receives enhanced usual care or to an enhanced usual care group plus structured SMBG (STG). A total of 504 patients will be enrolled; eligible patients at each site will be randomly selected using a defined protocol. Anticipated attrition of 20% will yield a sample size of at least 204 per arm, which will provide a 90% power to detect a difference of at least 0.5% in change from baseline in HbA1c values, assuming a common standard deviation of 1.5%. Differences in timing and degree of treatment intensification, cost effectiveness, and changes in patient self-management behaviours, mood, and quality of life (QOL) over time will also be assessed. Analysis of change in HbA1c and other dependent variables over time will be performed using both intent-to-treat and per protocol analyses. Trial results will be available in 2010.

**Discussion:**

The intervention and trial design builds upon previous research by emphasizing appropriate and collaborative use of SMBG by both patients and physicians. Utilization of per protocol and intent-to-treat analyses facilitates a comprehensive assessment of the intervention. Use of practice site cluster-randomisation reduces the potential for intervention contamination, and inclusion criteria (HbA1c ≥ 7.5%) reduces the possibility of floor effects. Inclusion of multiple dependent variables allows us to assess the broader impact of the intervention, including changes in patient and physician attitudes and behaviours.

**Trial Registration:**

Current Controlled Trials NCT00674986.

## Background

Over the past few decades, self-monitoring of blood glucose (SMBG) has been recognized as a core component of effective diabetes self-management [1-4]. This has been supported by a plethora of research that has consistently demonstrated that SMBG is a key contributor to good glycaemic control among insulin-using patients with type 1 (T1DM) [5,6] and type 2 diabetes (T2DM) [7-9]. It remains uncertain, however, whether SMBG is efficacious among the large number of T2DM patients who do not use insulin. Results to date have been decidedly mixed, with some studies pointing to significant glycaemic benefits resulting from SMBG use [10-14], while others have shown no significant benefits [15-18]. Given the costly nature of current T2DM care, especially as the worldwide prevalence of T2DM continues to grow rapidly, it is critical to determine whether resources devoted to SMBG are justified and being applied effectively. Therefore, using randomised controlled trial (RCT) methodology, this study seeks to test the effects of SMBG on metabolic outcomes in insulin naÏve T2DM patients, with special attention devoted to identifying those conditions under which SMBG is or is not beneficial.

This RCT is based on a comprehensive, critical review of the six largest RCTs that included insulin-naÏve T2DM patients [12-14,16-18] and published summaries of the literature [19-22]. Our review suggests that the inconsistent findings found in the literature to date may have resulted from problems in the actual SMBG intervention. These problems point to underlying concerns about the design of future studies, all of which have been raised recently by expert working groups [23,24]. If the actual benefit of SMBG in this population is to be determined definitively, careful attention must be given to these potential limitations; most importantly, we must be certain that the actual SMBG intervention itself is adequate, and that the study design permits a reasonable examination of the research question. Because our study builds upon the previous literature, we raise several questions about major research design and study implementation issues and then show how these issues are addressed in the new study.

### How adequate was the SMBG intervention?

SMBG is only one component of a larger diabetes management regimen. The potential value of SMBG lies in the subsequent actions which may result from its use, including actions that the patient makes directly (e.g., adjusting his/her dietary intake) and/or indirectly (e.g., sharing results with his/her healthcare provider (HCP), who may then recommend treatment changes). Without consideration of this context, efforts to assess any value associated with the simple act of blood glucose monitoring (e.g., the number of blood glucose tests/day) is relatively meaningless. Therefore, we view effective SMBG as a "package" of behaviours, a multi-component intervention that surrounds SMBG actions. In particular, an effective intervention must include three key elements: 1) recommended SMBG frequency and timing (is the prescribed testing regimen sufficiently frequent and comprehensive that meaningful and actionable glucose data can be obtained?); 2) patient response to SMBG results (do patients make use of their SMBG data appropriately?); and 3) HCP use of SMBG results (do HCPs view the SMBG results and make treatment adjustments as needed?). Many SMBG interventions reported in the literature were not comprehensive and were missing key components, thus limiting their potential impact on defined outcomes.

### What is optimal SMBG frequency and timing?

How many blood glucose tests are enough? Should testing take place at specified times during the day to account for postprandial spikes or the impact of other activities? Across the six RCTs, recommended SMBG frequency was 6 - 8 tests/week, with some but not all recommending postprandial testing. Assuming that the purpose is to collect enough data so that patients and/or their HCPs can see recognizable patterns and be confident about taking corrective action, is this frequency and timing sufficient? Most studies, however, do not provide a clear rationale to justify the frequency and timing of testing included in their protocols. Although there are no data that have established a clear threshold for optimal SMBG frequency, there is preliminary evidence suggesting that relatively intensive and structured SMBG, which includes preprandial and postprandial testing over three consecutive days, may be useful [2,23]. Furthermore, this form of intensive monitoring may make it easier for patients to see the glycaemic impact of their own actions, which can enhance their interest and willingness to engage in positive lifestyle changes.

### Do patients make use of SMBG results?

It is often assumed that SMBG functions like other feedback devices (e.g., pedometers) to promote corrective action. If patients do not use SMBG data appropriately, however, then the mere act of blood glucose monitoring may be pointless. Only three of the RCTs we reviewed [12-14] indicated that patients did interpret and respond to their SMBG results. For example, Guerci and colleagues [12] found that the percentage of patients following dietary recommendations remained constant over time in the SMBG arm and decreased significantly in the control arm, suggesting that maintaining dietary adherence was facilitated by the use of SMBG results. Thus, an effective SMBG intervention must include ways to assist patients in making effective use of SMBG data.

### Do HCPs make use of SMBG data?

A key value of SMBG is that it can provide HCPs with the information they need to make timely adjustments in medication and to make focused lifestyle recommendations. When this occurs, it can also help patients to see that their personal efforts to collect SMBG data are worthwhile, thus contributing to patients' ongoing motivation to continue SMBG over time. Nevertheless, two of the six RCTs [17,18] did not provide HCPs with access to SMBG data. Not surprisingly, these RCTs found that SMBG use was not associated with a significant improvement in glycaemic control. In contrast, several other studies provided HCPs with SMBG data to monitor and adjust therapy and as a basis for discussion with patients about their regimen [13,14]. A significant beneficial impact of SMBG was observed in these studies. In sum, these findings suggest that an effective SMBG intervention should include HCPs working closely and collaboratively with their patients, with SMBG data made available to both.

### What is the HbA1c level of patients accepted for study?

Statistically, it would be very difficult to demonstrate improvements in HbA1c due to any intervention if patients are already at or near their target HbA1c levels before the intervention began; there simply would be insufficient room for improvement to occur (floor effect). For example, in the DiGEM [16] trial, significant glycaemic improvement did not occur in any of the three study arms over the one-year period. However, mean HbA1c was 7.5% at baseline, which was at the upper limit of HbA1c targets for T2DM in the United Kingdom. Under such circumstances, neither patients nor HCPs may have been motivated to intensify therapy and, therefore, any favorable impact due to SMBG would have been difficult to demonstrate. It is noteworthy that mean baseline HbA1c was >8.0% in those RCTs that demonstrated a significant HbA1c improvement as a result of SMBG [12-14,17,18]. Therefore, sufficiently high HbA1c levels at baseline are required in RCTs to prevent floor effects from occurring.

### How can intervention contamination across study arms be avoided?

Randomisation of patients within a clinic or practice site was the most commonly used approach among the six RCTs. However, within-practice patient randomisation requires HCPs to become knowledgeable and experienced regarding both the experimental and control protocols, setting the stage for potential contamination, which may reduce differences between the arms of the trial. HCPs may unintentionally make use of the knowledge they acquire working with the intervention group patients to adjust their treatment of control group patients. To avoid this kind of contamination, many studies in primary care employ a cluster-randomisation strategy that randomises practices (not patients) to trial arms [23,24]. This strategy requires that a diverse set of practices is recruited and randomised to each arm. All patients within a given practice are then assigned to a single arm, thus preventing cross-arm contamination.

### Do patients complete the entire protocol as required?

If a substantial percentage of patients or HCPs do not complete the SMBG intervention protocol as required, it will be difficult to evaluate the effects of the intervention fairly. In three of the six RCTs (DiGEM, [16] Drew-King [17], and ESMON [18]) only 48%, 38.5%, and 65%, respectively, of patients completed the prescribed testing requirements. Most studies employed an intent-to-treat analysis to evaluate study findings, which does not account for how well patients actually complied with the study protocol, thus potentially leading to a faulty interpretation of the results. In situations like these, subsequent per protocol analyses may be useful to clarify the impact, if any, of the actual intervention and to explore the reasons why some patients regularly complied with the protocol and others did not. With this knowledge, it is possible to evaluate the effects of the completed intervention and to identify specific patient or disease-related characteristics that are associated with poor SMBG adherence. These can later be used to identify patient subgroups in need of special attention.

### Summary

Due to differences in SMBG intervention protocols and study designs, the value and utility of SMBG in insulin-naÏve T2DM has yet to be determined clearly. Major problems include: comprehensiveness of the prescribed SMBG intervention itself; lack of a rationale for recommended frequency and timing of SMBG; lack of utilization of SMBG findings by patient and HCPs; risk of intervention contamination that can limit between-group differences; floor effects that occur when patients have low levels of HbA1c at baseline; and the low levels of intervention adherence that can affect outcomes. Consequently, we have designed a study that attempts to address each of these problems directly, using a protocol that involves both patients and HCPs in a collaborative and comprehensive SMBG "package."

## Methods/Design

The Structured Testing Program (STeP) Study is a 12-month, cluster-randomised, multi-centre clinical trial to evaluate whether poorly controlled, non-insulin treated T2DM patients benefit from a comprehensive, integrated physician/patient intervention that uses structured SMBG. The study protocol was approved by the Copernicus Group (Central IRB) and is in compliance with the Helsinki Declaration [25].

The primary objective of the study is to determine if an integrated, intensive, and episodic SMBG intervention is associated with a significant reduction in HbA1c over 12 months, compared with enhanced usual care. The study will also examine group differences in: 1) timing and degree of treatment intensification; 2) changes in patient self-management behaviours over time; 3) changes in patient mood and quality of life (QOL) over time; and 4) cost effectiveness of the intervention.

### Hypothesis

The overarching hypothesis is that SMBG will lead to significant improvement in glycaemic control among non-insulin treated T2DM patients when it occurs frequently enough for the detection of actionable glucose patterns, and when patients and their physicians possess the knowledge, skills, and willingness to interpret and make use of SMBG results in a collaborative fashion. More specifically, we hypothesize that episodic, intensive SMBG - when used appropriately for enhancing patient understanding, promoting patient motivation, and initiating/adjusting therapy - will significantly improve long-term blood glucose control in people with poorly controlled (HbA1c ≥ 7.5%), non-insulin treated T2DM. The practical, yet comprehensive, SMBG protocol includes: 1) short periods of intensive SMBG, in which data are recorded on an easy-to-view, paper-and-pencil form, performed at least quarterly; 2) patient and physician training in the effective use of SMBG data; 3) patient presentation of their SMBG data to their physicians for regular review and discussion; and 4) physician review and discussion of SMBG data with their patients, followed by recommendations for therapy change (pharmacologic and non pharmacologic) as needed. The protocol places significant emphasis on the collaborative efforts and relationship between patients and their physicians because both play critical roles in the appropriate utilization of SMBG data.

### Subjects

Thirty-four primary care practice sites in the eastern United States will be identified and recruited for participation in the study. The sites will include both small and large primary care practices, which serve communities with a range of patient education, social class, and ethnicity that reflects the diversity of primary care settings in the US. Each site will then be randomly assigned (cluster-randomised design) via a defined protocol to enhanced usual care (active control group [ACG]) or enhanced usual care with structured testing (structured testing group [STG]). Additional sites may be recruited as needed to ensure enough patients (minimum of 504 enrolled patients) to adequately power the study.

Two hundred thirty-one patients will be enrolled in the ACG, and 273 in the STG, which we estimate will lead to a total of 204 patients completing each arm at 12 months. Differential enrollment of practices and patients will be undertaken initially to account for potential differences in rates of attrition between the two study arms. Each practice will generate a list of all potential study patients who meet age, diagnosis, and HbA1c inclusion criteria from their individual databases or chart review. Participating physicians will review the list and eliminate any subject for medical reasons, as required by many human research committees in the US. Remaining patients will then be randomly selected from the list, using an external, study-defined protocol.

Inclusion criteria are: T2DM for greater than one year that is managed by their primary care physician; age 25 or more years; HbA1c ≥ 7.5-12.0%; treated by diet, exercise, oral diabetes medication, and/or injectable incretin mimetic; read and write English without assistance; and has not participated in any other research protocol within the last 30 days. Exclusion criteria are: T1DM; managed with insulin at the start of study; C-peptide level greater than 0.50 ng/mL; under the care of an endocrinologist or diabetologist; used systemic oral or inhaled steroids more than 14 days within last 3 months; treated with chemotherapy or radiation therapy; plans to relocate or travel extensively during next year; pregnant or breast feeding; and severe depression or other severe psychological conditions.

### Design

**Figure 1:**
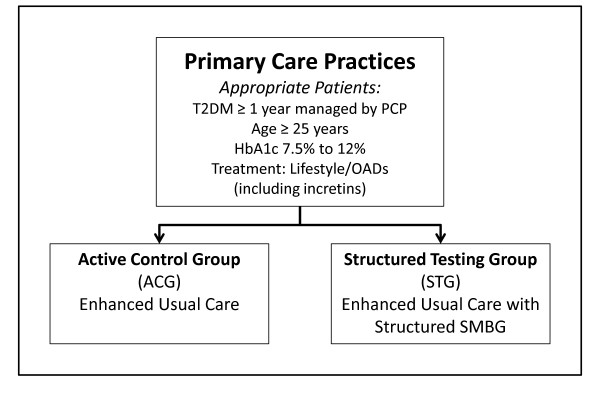
**Study design**.

The study design is shown in Figure 1. The STG arm will receive enhanced usual patient care plus a structured SMBG protocol at least quarterly. Patients in the ACG arm will visit their physicians quarterly and will continue their SMBG practice following their physician's recommendations. It is important to note that "usual care" in both groups involved more frequent clinic visits, point-of-care HbA1c tests, and free blood glucose meters and test strips; these additional services and resources are not generally available in most US primary care practices. Patients from both arms will be evaluated with the same scales and measures at the same time intervals over 12 months.

### Procedure

**Figure 2:**
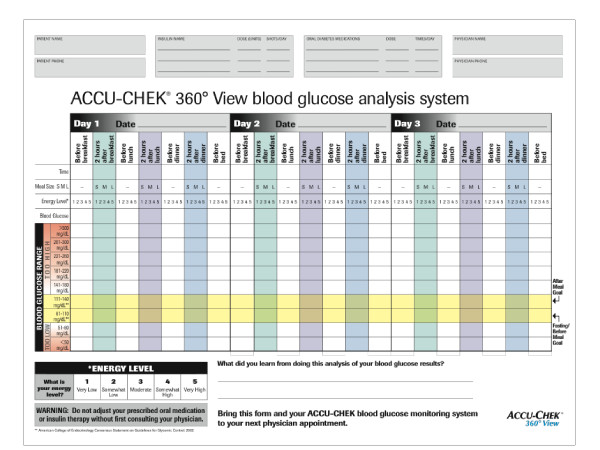
**Accu-Chek^® ^360° View blood glucose analysis system**.

STG participants will utilize the Accu-Chek^® ^360° View blood glucose analysis system (Tool) (Roche Diagnostics, Indianapolis, Indiana, USA). The system requires that patients record three consecutive day SMBG profiles. Each profile includes seven blood glucose tests/day (preprandial/postprandial at each meal and at bedtime), along with patient ratings of meal sizes and energy levels (see Figure 2). Space is provided to plot blood glucose results for better visualization of trends and patterns. Guidelines on the form indicate preprandial and postprandial blood glucose target ranges. The Tool also provides space for patients to note any comments or "learnings" relevant to their SMBG experiences. The Tool was designed to encourage collaborative discussion between patients and their physicians. Previous evaluations conducted with both patients and physicians have demonstrated that patients are willing and able to accurately complete the Tool [26] and that physicians are able to identify specific glucose patterns, determine the necessity for therapy change, and select specific pharmacological and lifestyle changes [26].

Physicians and staff in the ACG and STG arms will be informed about the clinical investigational plan, including the rationale for the study, design of the study protocol, subject-related procedures, and use of evaluation questionnaires. Patients in both arms will receive a blood glucose meter (Accu-Chek^® ^Aviva blood glucose meter system, Roche Diagnostics, Indianapolis, Indiana, USA) and will be thoroughly trained in its operation. ACG patients will be instructed to use their meters following their physician's recommendations.

STG physicians and staff will receive training on interpreting the SMBG data provided by the Tool and will be provided with an algorithm that describes various pharmacologic/lifestyle treatment strategies that can be utilized in response to the specific SMBG patterns identified in their analysis of the Tool. STG physicians will be contacted regularly over the 12 months of the study to enhance the consistency of the intervention over time. All physicians will be blinded to all study-collected measures and scales. Point-of-care HbA1c equipment will be provided to all practices to ensure that any differential availability of this equipment does not affect outcomes.

STG clinic staff will conduct one-on-one training sessions with each STG patient to teach the SMBG protocol. This includes instruction in the use of the meter and administration of a DVD instructional program to explain and demonstrate how to complete the Tool, interpret the SMBG data, and make appropriate changes in lifestyle treatments. One-on-one discussions between patients and staff will provide an opportunity to review the content and purpose of the SMBG intervention, discuss what was learned from the DVD, enhance general understanding, personalize SMBG activities, and address questions and concerns.

**Figure 3:**
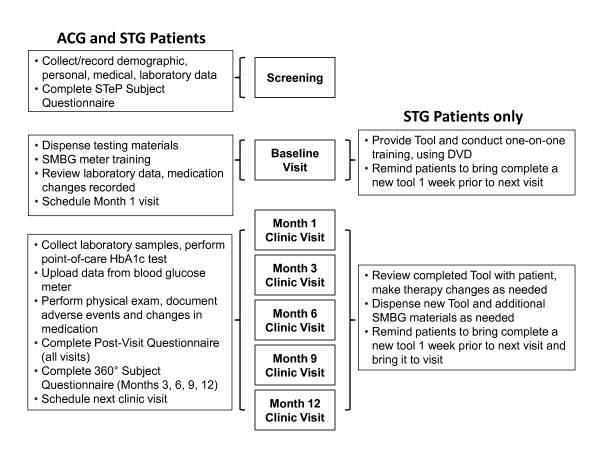
**Clinic visit schedule**.

Study duration will be 12 months, with patient visits occurring at initial screening and baseline, followed by completion of the Tool prior to visits at months 1, 3, 6, 9, and 12. The sequence of visits is described below and presented in Figure 3.

#### Screening visit

After eligible patients have been identified via study protocol and contacted by mail, they will be invited to attend an initial screening visit at their physician's office. At screening, investigators will describe the study in detail, obtain written informed consent, record demographics, collect relevant medical history/lifestyle activities, perform physical examinations, collect laboratory samples, and document all current medications, including vitamins and supplements. Patients will also complete the STeP questionnaire, which assesses disease management behaviour, mood, quality of life, and diabetes-related self-efficacy, using validated self-report instruments.

#### Baseline visit

At the baseline visit, which will occur within 14 days of screening, clinic staff will provide all patients with a blood glucose meter and test strips, and patients will be instructed in the use of their blood glucose monitoring system. Laboratory results from the screening visit will be reviewed to ensure inclusion criteria have been met; if not, subjects will be discontinued from the study. Any changes in medications, vitamins, and supplements will be recorded. No diabetes-related treatment changes will occur during the baseline visit.

Staff will provide STG patients with the Tool and conduct one-on-one training with each patient. The instructional DVD program will be viewed and discussed. STG patients will be asked to complete the Tool and bring it to the month 1 visit. ACG patients will not receive the Tool or any additional SMBG training.

#### Clinic visits (months 1, 3, 6, 9, and 12)

At subsequent visits, ACG and STG clinic staff will collect laboratory samples and a point-of-care HbA1c test will be performed. Adverse events and changes in medications (including vitamins and supplements) will be recorded and a brief physical examination will be performed. Physicians will document missed work days, changes in medical history and lifestyle activities. In addition, patients in both groups will bring in their meter so that data can be uploaded electronically. They also will report any changes they have made to their diabetes regimen since they were last seen. ACG and STG patients will complete the STeP questionnaire (months 3, 6, 9, and 12), as well as a post-visit questionnaire at each visit to assess patient satisfaction with the visit and to record patient reports of any physician recommendations for pharmacologic and/or lifestyle changes that occurred during the visit. STG patients also will be asked if they brought their 360 View SMBG Tool to the visit and if their physician reviewed the form with them.

In reviewing the Tool, areas of needed regimen change will be identified and treatment changes based on this information will be made jointly. Depending on the treatment changes recommended, STG patients may be asked to complete an additional Tool within the next few weeks to evaluate the effectiveness of the new treatment changes and to determine if additional changes are warranted. In all cases, another Tool will be provided to the STG patients at the end of each visit, to be completed during the week preceding each patient's next quarterly visit. One week prior to their next scheduled office visit, STG patients will be prompted via telephone by their physician's office to complete the Tool.

### Measures

#### Primary Endpoint

The primary endpoint is glycaemic control, as assessed by change in HbA1c from baseline over 12 months. Blood samples will be collected at screening and the scheduled clinic visits (months 3, 6, 9, and 12). HbA1c analysis will be conducted by a central laboratory (Covance, Indianapolis, Indiana, USA). All scales and measures will contain patient codes rather than patient identifiers. Patient identifiers will be restricted to their physician's and to senior investigators.

#### Secondary Endpoints

##### Treatment Intensification

Treatment intensification includes two components. The first is pharmacologic modification, which is defined as the initiation of a new medication, the increase or decrease in the dose of an existing medication, or the termination of an existing medication. The second is recommended lifestyle modification, which is defined as any change in diet, exercise, or other management approaches. The total number of visits with recommended medication or lifestyle modifications and the time to the first treatment change will be recorded for all subjects.

##### STeP Questionnaire

The STeP questionnaire incorporates the questions from standard psychometric instruments, as well as commonly used survey questions, to assess disease management behaviour, diabetes self-efficacy, well-being, depression, and diabetes-specific distress. Questions were drawn from the: 1) International Physical Activity Questionnaire (Short Form) [27]; 2) NCI Fruit & Vegetable Screener [28]: 3) National Cancer Institute (NCI) Percent Energy From Fat Screener [29]; 4) Hill-Bone Compliance to Medication Scale [30]; 5) Self-Monitoring of Blood Glucose (SMBG) [31]; 6) Treatment Self-Regulation Questionnaire (TSRQ) [32]; 7) Confidence in Diabetes Self-Care (CIDS-Type 2) [33]; 8) WHO-5 Well-Being Index (1998) [34]; 9) Diabetes Distress Scale (DDS) [35]; and 10) Patient Health Questionnaire 8 (PHQ-8) [36]. Clinic staff will be blinded from viewing these data.

##### Patient Post-Visit Survey

This survey records the subject's visit satisfaction and documents patient-reported treatment changes that were recommended by the physician during the visit. For STG patients, information on the usage of the Tool by both subject and physician will be collected.

##### Blood glucose meter data

SMBG data from both patient groups will be uploaded by the site coordinator directly to a web server at each study visit from the blood glucose meter via the Accu-Chek^® ^Smart Pix device (Roche Diagnostics, Indianapolis, Indiana, USA). Clinic staff will be blinded from viewing these data.

##### Clinical Data

Clinical laboratory evaluations will include hematolology, chemistry/immunology urinalysis, and point-of-care (HbA1c). Weight, height, blood pressure, pulse, respiration, and temperature will be recorded in the medical record and transcribed on the electronic record forms, where appropriate. A complete physical examination will be performed at screening and a brief physical examination will be performed at months 1, 3, 6, 9, and 12.

### Statistical analysis

In a cluster-randomised study, patients within a given cluster (in this case a clinical practice) often share many characteristics (e.g. ethnicity socioeconomic status, education level). Estimation of sample size, based on the assumption of the independence of individual observations, therefore may be inaccurate. Proper estimation of sample size for cluster-based studies requires estimates of the intra-class correlation coefficient (ICC) for each outcome variable of interest [37].

With change in HbA1c as the primary outcome variable, and using a two-sample t-test (two-sided, α = 0.05), a sample size of 204 per arm will have 90% power to detect a difference of at least 0.5% in change from baseline in HbA1c values, assuming a common standard deviation (SD) of 1.5%. The estimate of SD in HbA1c values was inflated from 1.15 to 1.50 due to the clustering effect [37,38].

#### Analysis of Dependent Variables

The analysis of change in HbA1c and other dependent variables (e.g. self-management behaviours) over time will be performed in two ways. First, the analysis will focus on the *intent-to-treat *(ITT) population, defined as all subjects who completed the baseline visit, regardless of their subsequent participation or compliance in the protocol over time. There will be no imputation of missing data values; however, a mechanism relevant to the missing data will be taken into account by using Linear Mixed Models (LMM) with SAS PROC MIXED, where it is assumed, initially, that the missing measurements of HbA1c are missing at random. Additional analyses of patient attrition at each step in the protocol from screening through the 12 month assessment also will be undertaken.

The second approach will be a *per protocol *(PP) analysis, which will compare ACG patients, STG patients who complied with the protocol, and STG patients who did not comply with the protocol. Compliance is defined as those STG patients who, at >4 clinic visits (out of a possible five visits), completed at least 80% of all blood glucose values on the Tool, brought their completed Tool to the clinic visit, and reported that their physicians looked at the Tool and discussed the results. This analysis will enable us to determine the effect of the SMBG intervention among those STG patients and physicians who participated fully in the program, compared to those who did not.

#### Additional Analyses (ITT and PP)

##### Mediator analyses

These analyses will examine whether critical variables, such as total number of visits, treatment intensification, physician recommendations for pharmacologic and/or lifestyle changes, frequency of Tool use, and changes in patient self-management behaviours (e.g., level of physical activity), mediated the effects of membership in each study arm on the primary and secondary outcomes over time.

##### Moderator analyses

These analyses will observe the effects of baseline patient and physician characteristics on subsequent outcomes over time. For example, we will explore the impact of baseline levels of patients' depressed mood, use of SMBG, number of medications, diabetes-related distress, and diabetes self-efficacy on outcomes. The impact of physician practice characteristics (e.g., years in practice, clinic size) will also be examined as they affect outcomes.

## Discussion

Given the growing worldwide diabetes epidemic, it is critical that resources devoted to diabetes management are applied effectively. Although the benefits of SMBG in T1DM and insulin treated T2DM are well-supported in the literature [5-9], the value and utility of SMBG in non-insulin-treated T2DM remains uncertain. Much of this uncertainty stems from notable differences among previous studies; most importantly, the adequacy of the study design to accurately measure the impact of SMBG and the adequacy of the SMBG intervention itself.

In designing the current study, we have attempted to address these limitations, with an emphasis on appropriate utilization of SMBG data by both patients and physicians working together. Building upon previous trials, our study addresses the value of SMBG as a method to enhance patient motivation and understanding of their disease, to modify patient behaviour in support of healthier lifestyles and to guide and support therapeutic changes by both physicians and patients. Furthermore, we have selected a large group of primary care practices to ensure a diverse patent population and have taken active steps to reduce a variety of potential biases through the use of randomisation procedures.

It is important to note that the intervention is not directed at just patient use of the paper Tool; rather, it involves both patient and physician behaviours and knowledge, which are impacted by the parallel tracks of intervention training. Moreover, the intervention emphasizes a collaborative relationship between patient and physician. Thus, we have conceptualized our SMBG intervention as not just the act of determining a blood glucose value; but rather, as both patients and physicians making appropriate use of SMBG data through informed treatment decisions. The results of this trial will be available in 2010.

## Competing interests

Funding for the study and preparation of the manuscript was provided by Roche Diagnostics, Indianapolis, Indiana, USA. WP, LF, CS, DH, CP, and ZJ serve as paid consultants to Roche Diagnostics for their involvement in the study design, study implementation and preparation of the manuscript. WHP has worked as a consultant for Roche Diagnostics, Abbott Diabetes Care (Alameda, California, USA), Amylin Pharmaceuticals (San Diego, California, USA), and Sanofi-Aventis (Bridgewater, New Jersey, USA). LF has worked as a consultant for Roche Diagnostics. CS has worked as a consultant for Roche Diagnostics, Abbott Laboratories, and Novartis Pharmaceuticals (Basel, Switzerland). DH has worked as a consultant for Roche Diagnostics, Abbott Diabetes Care, Amylin Pharmaceuticals, and Eli Lilly and Company (Indianapolis, Indiana, USA). CP has worked as a consultant for Roche Diagnostics, Abbott Diabetes Care, Amylin Pharmaceuticals, and Generex (Toronto, Ontario, Canada). ZJ has worked as a consultant for Roche Diagnostics. LA, MS and RW are employed by Roche Diagnostics.

## Authors' contributions

WP, LF, MS, and RW had the original idea for the study and wrote the study proposal and protocol. WP, LF, and RW developed study measures and intervention. DH, CS, and CP developed subject instructional materials and provided training. LA is the study site coordinator. ZJ provided statistical analysis support. WP, LF, and CP developed the manuscript, which was reviewed by CS, LA, ZJ, MS, and RW. All authors read and approved the final manuscript.

## Pre-publication history

The pre-publication history for this paper can be accessed here:

http://www.biomedcentral.com/1471-2296/11/37/prepub
